# Diabetes update: What's new, what's interesting

**DOI:** 10.1111/1753-0407.13308

**Published:** 2022-08-30

**Authors:** Zachary T. Bloomgarden

**Affiliations:** ^1^ Division of Endocrinology, Diabetes and Bone Disease, Department of Medicine Icahn School of Medicine at Mount Sinai New York USA

## 1 EPIDEMIOLOGY

How much exercise is the right amount? In a study of 1194 US adults with prediabetes and 493 with diabetes wearing an accelerometer for at least 1 day (mean 5.5) during the 2005–2006 National Health and Nutrition Examination Survey (NHANES), 200 and 138 had died over approximately 9 years of follow‐up, respectively. The mean number of steps per day was 8950 and 7151 and the 10th percentile of steps per day was 3779 and 2532 for the prediabetes and diabetes groups, respectively; the 10th percentile was associated with 4‐fold greater mortality risk than that of individuals walking >10 000 steps per day.^1^ The dilemma of interpretation of this observation is the question of whether a confounding bias exists, so that walking fewer steps per day applies to those individuals with other characteristics leading to greater mortality.^2^ The long follow‐up lessens but does not eliminate this concern, and it would be of interest to use techniques such as probabilistic bias analysis,^3^ or simply comparing characteristics of persons in the 10th percentile of steps per day with those in the overall group, to further address this issue.

Another study using the NHANES data set compared measured and predicted HbA1c (based on fasting and 2‐h post oral glucose) in 10 361 adults and 2201 youth to determine clinically significant mismatches; 15% of individuals age ≥65 and 7% of those aged 18–64 had measured HbA1c at least 0.5% over the predicted level, with this particularly an issue for non‐Hispanic Black persons, for whom 20% of those aged ≥18 had such a discrepancy,^4^ confirming earlier studies^5^ and reminding us that HbA1c is only an indirect measure of glycemia.

**FIGURE 1:**
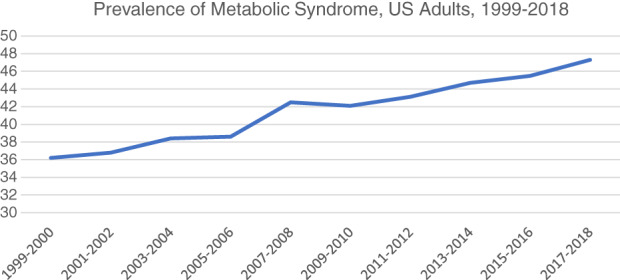
Prevalence of metabolic syndrome, US adults, 1999–2018, redrawn from supplemental data in Ref.^6^.

A study using NHANES data obtained from 55 081 US adults from 1999 to 2018 showed an increase in the proportion aged ≥65 from 15.85% to 20.4%, along with increases in obesity and diabetes; the prevalence of metabolic syndrome increased significantly from 36.2% to 47.3% (Figure 1).^6^ An analysis based on Global Burden of Disease 1990–2019 data showed near tripling of the number of deaths attributable to type 2 diabetes, from 0.61 to 1.47 million per year, along with an increase in disability‐associated life years from 25.48 to 66.30 million; the greatest increase has been in low‐middle income countries, with high body mass index (BMI) accounting for >40%, household and ambient air pollution accounting for >10%, low physical activity 7%–8%, and dietary factors approximately 6% of these increases, the latter particularly an issue in high‐income countries.^7^

A study from Finland reported changes in visual impairment due to diabetic retinopathy from 1980 to 2019; new visual impairment due to nonproliferative and proliferative diabetic retinopathy peaked in 1990–1999 at ~100 and ~40 cases per year per 100 000 persons with diabetes, respectively, declining to ~30 and ~20 in 2000–2009 and to <10 and 10 in 2010–2019, respectively, likely reflecting improved diabetes treatment along with improved screening for and treatment of retinopathy.^8^

## 2 THERAPY

Individual participant‐level meta‐analysis using data from 51 randomized clinical trials published between 1981 and 2014 involving 358 533 participants, 103 325 having type 2 diabetes at baseline, addressed the question of whether goals of blood pressure‐lowering treatment should be different for nondiabetic vs diabetic persons. The absolute risk reductions were similar for persons having vs. not having diabetes at 1.54% vs 1.61% for overall major cardiovascular events, 0.58% vs. 0.56% for stroke, 0.97% vs 0.91% for ischemic heart disease, and 0.77% vs 0.39% for heart failure, although reduction in cardiovascular (CV) mortality was significant (0.48%) for those not having diabetes but not for those with diabetes (0.09%). The optimal systolic blood pressure was <120 mm Hg both for persons having and not having diabetes, leading the authors to conclude, “In people with established type 2 diabetes, the current blood pressure thresholds for initiation of blood pressure treatment do not seem to be justified.”^9^ As a reminder, the current American Diabetes Association recommendations are for a target <140/90 mm Hg for individuals with diabetes and hypertension whose 10‐year CV disease risk is <15%, although “<130/80 mm Hg may be appropriate, if it can be safely attained” for those at higher CV risk.^10^

In analysis of outcome among persons with diabetes in the United Kingdom Clinical Practice Research Datalink, after weighting and adjustment for baseline confounders, 31 136 initiating insulin treatment with a long‐acting analog vs. 26 198 users of neutral protamine Hagedorn showed an 11% reduced risk of major CV events, 18% reduction in hospitalization for heart failure, and 10% reduction in CV mortality; although the authors acknowledge the potential that there might be “residual confounding by observable and non‐observable variables,” they suggest this might lead to greater use of the former classes of insulin.^11^

Given current recognition that the glucagon‐like peptide‐1 receptor activator class has not only glucose‐lowering but also cardioprotective benefit,^12^ it is important to clarify the effect of discontinuation of these agents. Among 327 persons having BMI >30 kg/m^2^ with at least one weight‐related comorbidity, but not having diabetes, treated with semaglutide 2.4 mg weekly vs. placebo for 68 weeks, 52 weeks after discontinuation of treatment the placebo group returned to baseline, and the semaglutide group, whose BMI had decreased from 37.6 to 31.2, had an increase in BMI to 35. Systolic and diastolic blood pressure, HbA1c, lipids, and C‐reactive protein all similarly returned toward or above baseline,^13^ suggesting that such treatment likely is required in an ongoing fashion for persons with diabetes as well.

An addition to CV prevention thought to be important has been the use of icosapent ethyl, which may reduce CV events in persons with CV disease having moderate or greater increase in triglyceride levels, particularly persons with diabetes.^14^ A recent analysis, in which investigators in the original study participated, suggests, however, that rather than icosapent ethyl being associated with benefit, the mineral oil comparator was associated with increases in total and oxidized low‐density lipoprotein cholesterol, C‐reactive protein, lipoprotein‐associated phospholipase A2, interleukin‐6, interleukin‐1β, and other potential mediators, both at 12‐ and at 24‐month follow‐up,^15^ leading this author to wonder whether the treatment is actually beneficial.

## References

[1] Del Pozo‐Cruz J , Alvarez‐Barbosa F , Gallardo‐Gomez D , Del Pozo Cruz B . Optimal number of steps per day to prevent all‐cause mortality in people with prediabetes and diabetes. Diabetes Care. 2022;dc220524. doi:10.2337/dc22-0524 35796565

[2] Luijendijk HJ , Page MJ , Burge H , Koolman X . Assessing risk of bias: a proposal for a unified framework for observational studies and randomized trials. BMC Med Res Methodol. 2020;20:237. doi:10.1186/s12874-020-01115-7 32967622 PMC7510067

[3] Hunnicutt JN , Ulbricht CM , Chrysanthopoulou SA , Lapane KL . Probabilistic bias analysis in pharmacoepidemiology and comparative effectiveness research: a systematic review. Pharmacoepidemiol Drug Saf. 2016;25:1343‐1353. doi:10.1002/pds.4076 27593968 PMC5272921

[4] Staimez LR , Kipling LM , Nina Ham J , et al. Potential misclassification of diabetes and prediabetes in the U.S.: mismatched HbA1c and glucose in NHANES 2005–2016. Diabetes Res Clin Pract. 2022;189:109935. doi:10.1016/j.diabres.2022.109935 35662612 PMC10148706

[5] Misra A , Bloomgarden ZT . Discordance between HbA1c and glycemia. J Diabetes. 2018 Dec;10(12):908‐910. 30126041 10.1111/1753-0407.12843

[6] O'Hearn M , Lauren BN , Wong JB , Kim DD , Mozaffarian D . Trends and disparities in cardiometabolic health among U.S. adults, 1999–2018. J Am Coll Cardiol. 2022;80(2):138‐151. doi:10.1016/j.jacc.2022.04.046 35798448 PMC10475326

[7] Liu J , Bai R , Chai Z , Cooper ME , Zimmet PZ , Zhang L . Low‐ and middle‐income countries demonstrate rapid growth of type 2 diabetes: an analysis based on Global Burden of Disease 1990–2019 data. Diabetologia. 2022;65:1339‐1352. doi:10.1007/s00125-022-05713-6 35587275 PMC9118183

[8] Purola PKM , Ojamo MUI , Gissler M , Uusitalo HMT . Changes in visual impairment due to diabetic retinopathy during 1980–2019 based on nationwide register data. Diabetes Care. 2022;dc212369. doi:10.2337/dc21-2369 35838317 PMC9472510

[9] Nazarzadeh M , Bidel Z , Canoy D , et al. Blood pressure‐lowering treatment for prevention of major cardiovascular diseases in people with and without type 2 diabetes: an individual participant‐level data meta‐analysis. Lancet Diabetes Endocrinol. 2022. doi:10.1016/S2213-8587(22)00172-3 PMC9622419 35878651

[10] American Diabetes Association Professional Practice Committee . Cardiovascular Disease and Risk Management: Standards of Medical Care in Diabetes‐2022. Diabetes Care. 2022;45(Suppl 1):S144‐S174. doi:10.2337/dc22-S010 34964815

[11] Brunetti VC , Yun Yu OH , Platt RW , Filion KB . The association of long‐acting insulin analogue use versus neutral protamine Hagedorn insulin use and the risk of major adverse cardiovascular events among individuals with type 2 diabetes: a population‐based cohort study. Diabetes Obes Metab. doi:10.1111/dom.14802 35726454

[12] Marx N , Davies MJ , Grant PJ , et al. Guideline recommendations and the positioning of newer drugs in type 2 diabetes care. Lancet Diabetes Endocrinol. 2021;9(1):46‐52. doi:10.1016/S2213-8587(20)30343-0 33159841 PMC12140926

[13] Wilding JPH , Batterham RL , Davies M , et al. Weight regain and cardiometabolic effects after withdrawal of semaglutide: the STEP 1 trial extension. Diabetes Obes Metab. 2022;24(8):1553‐1564. doi:10.1111/dom.14725 35441470 PMC9542252

[14] Bhatt DL , Steg PG , Miller M , et al. Cardiovascular risk reduction with icosapent ethyl for hypertriglyceridemia. N Engl J Med. 2019;380(1):11‐22. 30415628 10.1056/NEJMoa1812792

[15] Ridker PM , Rifai N , MacFadyen J , et al. Effects of randomized treatment with icosapent ethyl and a mineral oil comparator on interleukin‐1β, interleukin‐6, C‐reactive protein, oxidized low‐density lipoprotein cholesterol, homocysteine, lipoprotein(a), and lipoprotein‐associated phospholipase A_2_: a REDUCE‐IT biomarker substudy. Circulation. 2022;146(5):372‐379. doi:10.1161/CIRCULATIONAHA.122.059410 35762321

